# School-based interventions for sexual education and sexual violence prevention in adolescence: systematic review

**DOI:** 10.1186/s41155-026-00390-5

**Published:** 2026-04-18

**Authors:** Kathyllen Bezerra dos Santos, Vanessa Barbosa Romera Leme, Ana Carolina Fagundes dos Santos, Sheila Giardini Murta

**Affiliations:** 1 Rio de Janeiro State University, Rio de Janeiro, Brazil; 2 University of Brasília, Brasília, Brazil

**Keywords:** Sex education, Sexual violence prevention, Adolescence, High school

## Abstract

**Background:**

Sexual violence against adolescents constitutes a serious violation of human rights, with negative repercussions on the physical, emotional, and social development of this population. Comprehensive Sexuality Education interventions have proven to be promising strategies for preventing violence in school contexts, as they promote adolescent empowerment and recognize sexuality as a fundamental dimension of human development.

**Objective:**

This systematic review examined interventions conducted with high school adolescents in school contexts, focusing on comprehensive sexuality education and the universal prevention of sexual violence in adolescence.

**Methods:**

The PRISMA guidelines and PICOS criteria were used to identify, select, evaluate, and synthesize the included studies. The databases searched were SciELO, Virtual Health Library, Web of Science, PsycINFO, DOAJ and PubMed. Risk of bias was systematically assessed using design-specific tools according to study type, including the Standards for Reporting Qualitative Research for qualitative studies, ROBUST for observational designs, RoB 2 for randomized trials, and ROBINS-I for non-randomized interventions, ensuring methodological rigor and comparability across study designs.

**Results:**

A total of 15 studies published between 2019 and 2024 were identified through electronic databases. The findings indicated that interventions were most effective in increasing knowledge about sexually transmitted infections, contraceptive methods, and consent. However, low consistency was observed in translating such knowledge into behavioral change, with limited outcomes in improving protective practices and effectively reducing indicators of sexual violence. The main limitations involved the use of convenience samples, lack of follow-up, absence of analyses considering the intersectionality of race, gender, and sexuality, and the exclusion of content related to LGBTQIAPN+ populations.

**Conclusion:**

The findings of this review reveal limitations stemming from the heterogeneity of methodological designs, restricting the potential for broader generalizations. While the studies demonstrated efficacy in knowledge promotion, limitations were observed regarding the effective prevention of sexual violence against adolescents, possibly related to the evaluation measures employed and the stigmatized nature of the topics addressed. Future research should adopt more robust methodologies, include longitudinal follow-ups of interventions, and engage social actors from the context to develop content that is more socially relevant and tailored to participants’ needs.

**Supplementary Information:**

The online version contains supplementary material available at 10.1186/s41155-026-00390-5.

## Introduction

From a global human rights and public health perspective, protecting children and adolescents from all forms of violence constitutes a fundamental ethical and political imperative. The World Health Organization (WHO, 2002) defines sexual violence as any sexual act or attempt, including unwanted comments and advances, involving coercion or abuse of power, perpetrated by known or unknown individuals in family and institutional contexts. Evidence indicates that the consequences of sexual violence during childhood and adolescence extend into adulthood and are associated with risky sexual behaviors, post-traumatic stress disorder, substance abuse, body image distortions, and depression (Hailes et al., 2019). These findings reinforce the urgency of preventive strategies grounded in social, legislative, and educational public policies that ensure the effectiveness of already established rights (Mendonça & Lima, 2023; WHO, 2002).

Within this global framework, Brazil represents a paradigmatic case of how international human rights and public health guidelines are operationalized at the national level. In alignment with these principles, the Brazilian Child and Adolescent Statute (Brazil, 1990) establishes the doctrine of full protection, guaranteeing children and adolescents the right to be safeguarded from all forms of violence, including sexual violence (Silva & Alberto, 2022). The 1990s marked advances in the visibility of sexual violence in Brazil, enabling the development of specific public policies (Mendonça & Lima, 2023). The approval of the National Plan to Combat Sexual Violence Against Children and Adolescents [Plano Nacional de Enfrentamento à Violência Sexual contra Crianças e Adolescentes] in 2000 by the National Council for the Rights of Children and Adolescents [Conselho Nacional dos Direitos da Criança e do Adolescente] represented a milestone by promoting the creation of intervention methodologies (Campos & Urnau, 2021).

Despite this, the data remain alarming: globally, one in two children and adolescents aged 2 to 17 experiences some form of violence each year, and around 120 million girls have experienced forced sexual contact before the age of 20 (WHO, 2020). In Brazil, between 2017 and 2020, 179,277 cases of rape or statutory rape among victims aged 0–19 were reported (United Nations Children’s Fund [UNICEF] & Brazilian Public Security Forum, 2021). Gender inequalities are particularly striking: about 80% of the victims are girls, mostly between 10 and 14 years old, and among adolescents over 15, more than 90% of cases involve girls (UNICEF & Brazilian Public Security Forum, 2021).

These figures likely reflect substantial underreporting, given the difficulties in monitoring and the lack of data on key variables such as age, race/ethnicity, and social context (UNICEF & Brazilian Public Security Forum, 2021). Most cases occur in domestic environments, with perpetrators predominantly known to the victims, such as fathers (15.7%), mothers (15.9%), boyfriends (6.2%), spouses (4.4%), or other acquaintances (20.2%), while only 13.2% of perpetrators are strangers (Malta et al., 2025). Early sexual victimization also increases the likelihood of involvement in cycles of intimate violence in adulthood, both as victims and as perpetrators, thereby reinforcing the intergenerational nature of the phenomenon (WHO, 2020).

From a psychological perspective, adolescence represents a critical developmental period characterized by heightened sensitivity to peer norms, identity exploration, and ongoing emotional and relational maturation, all of which shape vulnerability to both dating and sexual violence, as well as responsiveness to preventive interventions (Moraes et al., 2025; Van de Bongardt et al., 2018; WHO, 2024). Evidence from social and health psychology indicates that competencies such as social self-efficacy, assertiveness, and emotion regulation function as key protective factors, whereas adherence to rigid gender norms, limited sexual communication, and the normative acceptance of coercion operate as risk factors (Agu et al., 2022; Brasileiro et al., 2021; Moraes et al., 2025).

Within this context, educational institutions play a strategic role in prevention. The United Nations Educational, Scientific and Cultural Organization (UNESCO, 2020) underscores that Comprehensive Sexuality Education (CSE) interventions are essential to fostering youth empowerment and addressing gender inequalities and sexual violence. CSE and sexual violence prevention are conceptually interdependent. Key components of CSE, such as consent education, privacy, refusal and negotiation skills, critical reflection on gender norms, and recognition of coercive dynamics, directly target mechanisms associated with sexual victimization and perpetration (UNESCO, 2019).

In Brazil, the inclusion of topics related to sexuality was legitimized in the National Curriculum Parameters [Parâmetros Curriculares Nacionais] (PCN) in the late 1990s, which incorporated Sexual Education, currently understood as CSE, as a cross-cutting and progressive theme by recognizing sexuality as a constitutive dimension of human experience, moving beyond strictly biomedical or hygienist perspectives (Cassiavillani & Albrecht, 2023; Souza et al., 2019). However, conservative resistance, such as the Escola sem Partido [School Without Party] movement, has challenged the implementation of CSE, reinforcing moralistic and misinformed views about sexuality (Mark & Wu, 2022; Mendonça & Lima, 2023; Souza et al., 2019). The failure to promote sexual rights, in turn, constitutes a form of structural violence, depriving adolescents of information that could break cycles of victimization (Campos & Urnau, 2021).

The scientific literature, however, reveals important gaps: systematic reviews on the school context generally address either CSE (Niland et al., 2024) or sexual violence (Che Yusof et al., 2022) separately, neglecting their intersection. A study conducted in Italy showed that only 33% to 42% of its regions included sexual violence within CSE interventions (Lo Moro et al., 2023). A review carried out in Brazil highlighted a narrow focus on the prevention of STIs/HIV and adolescent pregnancy, without addressing sexual violence (Furlanetto et al., 2018). To our knowledge, no prior systematic review has simultaneously examined school-based CSE and sexual violence prevention during adolescence. Previous reviews addressed these domains separately (Che Yusof et al., 2022; Furlanetto et al., 2018; Niland et al., 2024). Considering this scenario, the present systematic review aims to integrate the dimensions of CSE and universal prevention of sexual violence in school settings, drawing on recent scientific evidence. Universal prevention was proposed by Gordon (1983), who defined it as a set of measures in which the benefits outweigh the associated costs and risks and are therefore desirable for the entire population. In the field of sexual violence prevention among adolescents, this framework supports the implementation of school-based interventions targeting all students, regardless of their individual risk status.

Most previous reviews have examined school-based interventions across broad age ranges, often combining early and middle adolescence, which has limited more fine-grained analyses of upper secondary students as a specific target population (Che Yusof et al., 2022; Niland et al., 2024). Upper secondary education therefore constitutes a privileged stage for this investigation, given adolescents’ increased cognitive autonomy, exposure to social and sexual risks, and the concentration of educational policies targeting this age group (UNESCO, 2019). CSE interventions aim not merely to convey information, but to also activate psychological mechanisms of change by fostering cognitive, socioemotional, and relational competencies that underpin sexual decision-making, consent, and help-seeking (Goldfarb & Lieberman, 2021; UNESCO, 2019). These constructs enhance the analytical depth of the review and clarify how psychologically informed approaches can contribute to the prevention of sexual violence.

In this review, we propose a descriptive mapping of intervention characteristics as an analytical strategy to identify the structural, methodological, and conceptual gaps that hinder the integration of CSE and sexual violence prevention. By systematizing how interventions have been implemented, specifically concerning their theoretical frameworks, target populations, content, and outcomes, this review aims to generate evidence that can inform the development of more integrated and context-sensitive educational practices.

Accordingly, the guiding question of this review was: How have school-based interventions with upper secondary students been implemented, articulating CSE and the universal prevention of sexual violence? By addressing this question, this study seeks to contribute to the promotion of more integrated, evidence-based educational practices aligned with the protection of adolescents’ sexual and reproductive rights. Thus, this systematic review examined school-based interventions targeting upper secondary students, focused on CSE and the universal prevention of sexual violence during adolescence. Interventions were included if they explicitly addressed sexual violence or incorporated content conceptually related to its prevention, such as consent, coercion, harassment and negotiation skills, even when sexual violence was not the primary or explicit focus of the intervention.

## Method

This systematic review was conducted following the criteria established by the PRISMA protocol (Page et al., 2021) and guided by the PICOS framework, which structures the formulation of the research question and the definition of eligibility criteria in a systematic and transparent manner. The components considered were: P (Participants) - adolescents enrolled in upper secondary education; I (Intervention) - programs focused on CSE and the prevention of sexual violence; C (Comparison) - groups that did not receive interventions related to CSE or sexual violence prevention; O (Outcomes) - evaluation of participants’ perceptions regarding the addressed topics through indicators such as knowledge, attitudes, self-efficacy, intentions, and/or behaviors, as well as data on the implementation or results of the interventions; and S (Study designs) - empirical studies employing quantitative and/or qualitative designs (Eriksen & Frandsen, 2018). This methodological approach supports the development of clearly defined research questions and the refinement of the search strategy, ensuring greater rigor and accuracy in the selection of included studies. No a priori hierarchy of outcomes was defined, as the primary objective of this review was to comprehensively map the breadth of effects reported across interventions.

In alignment with the 2030 Agenda for Sustainable Development Goals, the United Nations (UN) launched in 2016 the global campaign End Violence Against Children, whose central aim is to reduce and eradicate all forms of violence, including sexual violence, against children and adolescents (WHO, 2016). Brazil formally joined the initiative in 2018, and the following year UNESCO (2019) published the International Technical Guidance on Sexuality Education, a reference document that emphasizes the importance of evidence-based CSE aimed at promoting sexual and reproductive health. This international milestone has mobilized governments and organizations to review and update their CSE interventions in line with global guidelines. Within this context, the year 2019 was defined as the temporal cutoff for this systematic review, as it represents a strategic turning point in policies and practices related to CSE and concentrates the most recent studies that integrate the themes of sexuality and the prevention of sexual violence in adolescence.

The following eligibility criteria were adopted for the inclusion of articles: (1) written in Portuguese, English, or Spanish; (2) original empirical studies; (3) peer-reviewed; (4) available in full-text format; (5) involving interventions focused on CSE that explicitly addressed sexual violence prevention or incorporated content conceptually related to its prevention, such as consent, coercion, harassment, or negotiation skills, even when sexual violence was not the central focus of the intervention; (6) implemented in school settings, whether public or private, involving students enrolled in upper secondary education; and (7) using qualitative, quantitative, and/or mixed-method designs.

The exclusion criteria were as follows: (1) studies classified as grey literature or those using secondary data, such as reviews, meta-analyses, and theoretical studies; (2) studies examining perceptions of CSE and sexual violence prevention outside the school setting, such as within the family environment; (3) interventions with adolescents enrolled in other educational levels, such as primary or higher education; and (4) studies involving adolescents with intellectual disabilities and/or neurodevelopmental disorders, because these populations require specialized pedagogical adaptations and represent a distinct literature on disability-inclusive CSE.

This systematic review was conducted following registration on PROSPERO (International prospective register of systematic reviews), under registration number CRD42024602478. The search for studies was carried out between October and November 2024 by two independent reviewers using a blinded assessment process. The screening team consisted of two doctoral-level psychology students with prior training and experience in conducting systematic reviews. All reviewers had academic backgrounds in public health, psychology, and education, as well as experience in research on CSE and violence prevention. Discrepancies in study selection and data extraction were resolved through discussion and, when necessary, adjudicated by a senior researcher, a PhD in Psychology with extensive experience in systematic reviews.

Six databases were selected: SciELO (Scientific Electronic Library Online), Biblioteca Virtual em Saúde (BVS) [Virtual Health Library], Web of Science, PsycINFO, PubMed and DOAJ (Directory of Open Access Journals). Searches in English were conducted across all these databases, whereas searches in Portuguese were restricted to SciELO and BVS. Access to full-text articles was obtained through the CAPES journal platform, using institutional access provided by the researchers’ affiliated universities. Thus, the studies included in this review were not limited to open-access publications but were selected according to the availability of full texts through these academic databases. The choice of databases and combinations of English keywords was informed by an exploratory review of systematic reviews addressing, at least in part, CSE and sexual violence (Carvalho et al., 2024; Che Yusof et al., 2022; Niland et al., 2024). The final search was conducted on November 11, 2024.

Multiple combinations of search terms were pilot-tested in each database to optimize retrieval. The final search strings were selected based on their ability to yield the highest number of articles aligned with the conceptual scope of the review. The following combination of keywords was used: (“sex education”) OR (“sexual violence prevention”) OR (“sexual abuse prevention”) AND (promotion OR intervention) AND (school). The Portuguese search strategy remained consistent throughout the process, using the following terms: (“educação sexual” OR “prevenção sexual” OR “prevenção de violência sexual” OR “prevenção de abuso sexual” OR “prevenção de agressão sexual”) AND (promoção OR intervenção) AND (escola OR “ensino médio”) AND (adolescente OR jovem). When applicable, filters were used to include only peer-reviewed full-text articles. The complete search strategies for each database are presented in Appendix A.

**Fig. 1:**
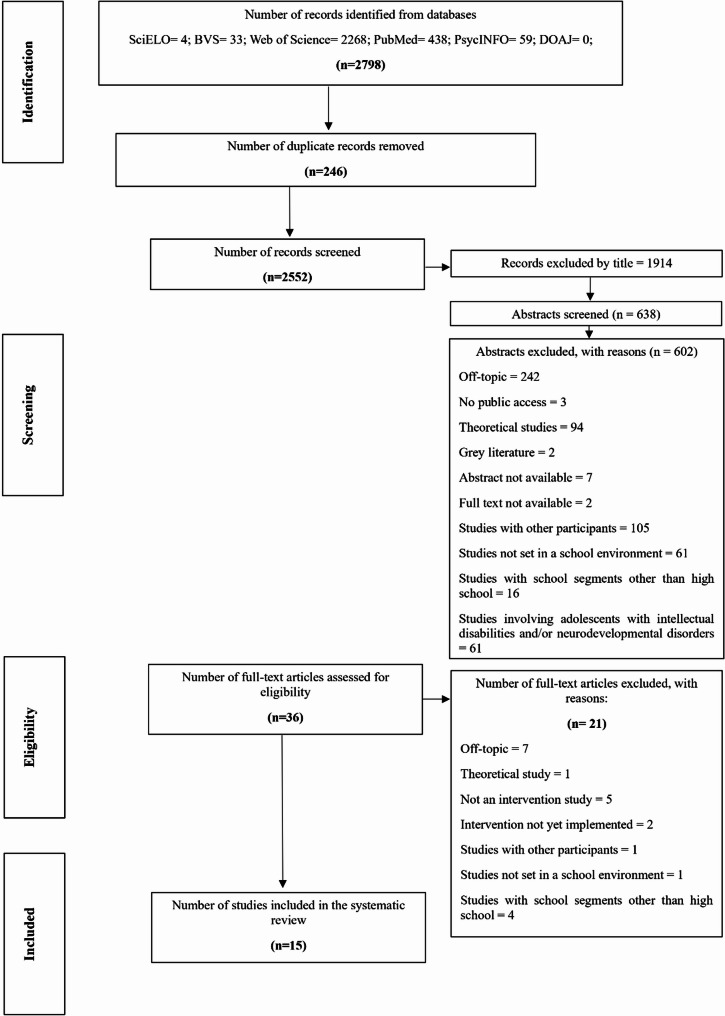
Flowchart of the systematic literature review

All references retrieved from the databases were exported and collated into a single Microsoft Excel spreadsheet specifically designed to manage the screening process. Duplicate records were removed using a two-step procedure: automated detection through Excel’s built-in matching functions, followed by manual verification to ensure accuracy and prevent the inadvertent exclusion of unique entries. Following the PRISMA guidelines (Page et al., 2021), data extraction was performed independently by each reviewer. First, titles and abstracts were screened, followed by full-text reading to assess eligibility (Fig. 1). All potentially eligible articles were read in full to determine their final inclusion. Interrater agreement regarding article eligibility was assessed using Cohen’s Kappa, (Kappa = 0.655, with 82.9% concordant decisions; Landis & Koch, 1977), indicating moderate-to-substantial agreement. This value, while robust, reflects the inherent complexity of the eligibility assessment process, given the conceptual heterogeneity of the interventions and the diversity of the outcome domains under review.

Data analysis was conducted according to the following criteria: (1) the country where the study was conducted, to examine cultural differences and identify regions with higher research output; (2) the theoretical framework underpinning the study, to identify the most prominent theoretical foundations; (3) the participants’ profiles, including age and gender, to determine whether the studies targeted specific genders or were more inclusive; (4) the instruments used to assess knowledge regarding CSE and/or sexual violence; (5) the study design, to explore whether methodological approaches influenced the findings, acknowledging that multiple designs were included in this review; (6) the main outcomes; and (7) reported limitations and/or future research agendas, to identify gaps to be addressed by subsequent studies.

The included studies were also assessed for risk of bias. Qualitative studies were evaluated using the Standards for Reporting Qualitative Research (O’Brien et al., 2014), which assigns a maximum score of 21 points, with higher scores indicating lower risk of bias. Quantitative survey or evaluation studies were assessed using the Risk of Bias Utilized for Surveys Tool (ROBUST) (Nudelman & Otto, 2020), which applies a numerical scale based on eight items, where higher scores indicate lower risk of bias. Randomized controlled trials were assessed using the RoB 2 (Revised Tool to Assess Risk of Bias in Randomized Trials; Sterne et al., 2019), which provides a qualitative classification across five domains as “low risk,” “some concerns,” or “high risk.” Non-randomized intervention studies were assessed using the ROBINS-I (Risk Of Bias In Non-randomized Studies of Interventions; Sterne et al., 2016), which also provides a qualitative rating across seven domains as “low risk,” “moderate risk,” “serious risk,” or “critical risk.” Results from the different risk-of-bias tools were not aggregated into a single score, instead, they informed a qualitative assessment of evidence strength across study designs. Bias assessments and outcome evaluations were completed independently by two reviewers. In addition, potential conflicts of interest and funding-related bias were assessed through systematic examination of the funding statements, conflict-of-interest disclosures, and acknowledgments sections of all included studies. No explicit conflicts of interest or indications of funding-related bias were identified.

## Results

The keyword search across the selected databases yielded 2,798 studies. Of these, 246 were excluded due to duplication, leaving 2,552 records for screening. During title screening, 1,914 articles were excluded for being off-topic, resulting in 638 abstracts for evaluation. After reviewing the abstracts, 602 studies were excluded for not meeting the objectives of this review. As a result, 36 studies were selected for full-text reading. The assessment of abstracts and full texts, based on the predefined eligibility criteria, resulted in the inclusion of 15 studies in the database. A peak in publications occurred in 2022, with five articles published that year (Berutich et al., 2022; Gómez-Lugo et al., 2022; Millanzi et al., 2022; Nuttall et al., 2022; Rasberry et al., 2022). Table 1 presents the main characteristics of the studies included in this review. The table is structured according to the guidelines proposed by Hoffmann et al. (2014) for reporting interventions, as well as formats adopted in high-impact systematic reviews (Garon-Carrier et al., 2024). This structure facilitated a comprehensive description and a quality assessment of the interventions.

**Table 1:** Main characteristics of the included studies (*N* = 15)

Authors and Country	Study Design	Topic/Focus	Knowledge Assessment Instrument on CSE and/or Sexual Violence	Study Participants	Main Results	Limitations/Research Agenda	Risk of Bias Assessment (Tool Used)
Agu et al. (2024) Nigeria	Quasi-experimental evaluation/survey study design	School-based sexual and reproductive health intervention to understand the needs of youth.	WHO Illustrative Questionnaire (Cleland, 2001).	Adolescents aged 13–18 (*n* = 514). Also included for training: state instructors (*n* = 29); school staff (*n* = 22), and peer mentors (*n* = 22). Community leaders (*n* = 20) from each selected community (*n* = 4) were engaged.	Most adolescents perceived a need for sexual and reproductive health information, primarily concerning condoms and STIs. Adolescents in the intervention group were more likely to perceive a need for information on these issues, indicating the effectiveness of targeted interventions. However, within this same group, there was a lower perceived need for information on puberty and STIs, which may have resulted from the awareness raised by the interventions.	The findings cannot be generalized, as they reflect information provided specifically to adolescents in a school setting, excluding out-of-school youth. Furthermore, the study relied solely on quantitative methods, limiting a deeper exploration of the topic. It is noted that due to the sensitive nature of sexuality, there is a risk of social desirability bias in participant responses.	Score = 6 Moderate risk (ROBUST)
Berutich et al. (2022) Spain	Observational descriptive evaluation/survey study design	To assess whether different levels of intervention within the Forma Joven Program lead to differences in knowledge and attitudes towards sexuality among students.	Questionnaire on attitudes and knowledge about sexuality, adapted from Barella Balboa (2002).	Adolescents (*n* = 1237) with varying levels of exposure to counseling/intervention sessions.	The study found that in some areas of the questionnaire, a higher number of counseling sessions was associated with greater knowledge and more positive attitudes among students. It highlights that the results likely depend more on the quality rather than the quantity of the sessions. Participants demonstrated good knowledge of condom use, despite existing misinformation regarding their correct use. Alarmingly, 6% of respondents believed that a person can be forced to have sex against their will.	No specific limitations were noted. The authors recommend developing materials to assist professionals in delivering content during interventions and conducting a study on the quality of educational interventions on sexuality in schools.	Score = 4 Moderate risk (ROBUST)
Edwards et al. (2023) USA	Quantitative, quasi-experimental evaluation study design.	The diffusion effects of a youth-led sexual violence prevention intervention (Youth VIP).	Questionnaire addressing proactive and reactive bystander behaviors related to sexual violence. Additionally, four items from Cook-Craig et al. (2014) assessing sexual coercion were used.	Adolescents (*n* = 1172) who had not participated in Youth Voices in Prevention (Youth VIP) activities.	Increased communication about Youth VIP among students was noted because of program participation. Furthermore, youth with friends who participated in the program were more likely to report hearing their friends talk about Youth VIP and to discuss it more with their own friends, compared to youth unconnected to other Youth VIP participants.	No significant diffusion effects on behaviors were found. Despite the large overall sample, the number of students who participated in Youth VIP events or heard about the activities was relatively small. The study was also unable to capture whether student evaluations of the activities were positive, negative, or neutral. Additionally, although a longitudinal design was used, the follow-up period was not long enough.	Score = 5 Moderate risk (ROBUST)
Gómez‑Lugo et al. (2022) Colombia	Quantitative, randomized controlled trial (RCT) design.	Efficacy evaluation of the COMPAS Program (Competencies for Adolescents with Healthy Sexuality) in promoting healthy sexual behaviors and reducing sexual risks among adolescents.	Individual questionnaires developed by the authors assessing sexual behaviors and predictors of safe sex, alongside adapted scales including Knowledge Scale on STIs and HIV (Espada et al.,^ 2014^; Abello-Luque et al., 2021), HIV-Related Attitudes Scale (Gómez-Lugo et al., 2022), and Sexual Assertiveness Scale (Vallejo-Medina et al., 2017).	Adolescents aged 12 to 19 (*n* = 2047).	The results were positive. The initial hypothesis of short-term efficacy was supported, as the experimental group showed improvement in variables (risky sexual behaviors, knowledge about STIs and HIV, self-efficacy, and sexual assertiveness) compared to the control group, which was maintained at the six-month follow-up. However, there was a reduction in the intention to use condoms, highlighting the need for regular CSE to achieve long-term change. Compared to the Spanish version, the program was unable to modify the normative perception of condom use.	The results cannot be generalized due to the sample size and the high dropout rate throughout the program. Sociodemographic differences are also noted, and as most adolescents identified as heterosexual, the efficacy for LGBTQIAPN+ adolescents could not be determined. Future research should include a long-term evaluation of the program and test its efficacy in more vulnerable populations.	High risk (RoB 2)
May et al. (2021) United Kingdom	Qualitative study with a phenomenological approach.	School-based theater education program for raising awareness about Child and Adolescent Sexual Exploitation.	Semi-structured interviews with adolescents conducted in a focus group format. The interviews addressed questions such as “What do young people know now that they didn’t know before?“, aiming to assess the knowledge acquired after participation in the program.	Adolescents aged 13 to 15 (*n* = 19).	Participants in the school-based theater education program developed awareness and knowledge about child sexual exploitation and abuse. This included aspects related to victims, perpetrators, harmful and abusive relationships, as well as how to act and avoid harm if something occurs.	Participants were self-selected volunteers, potentially resulting in a sample more engaged with the topic. A small representation of male participants, preventing gender-based comparisons. Furthermore, participants with disabilities were not included.	Score = 17 (Standards for reporting qualitative)
Meiksin et al. (2020) United Kingdom	Quantitative, randomized controlled trial (RCT) pilot study design.	Pilot study of a school-based relationships and CSE intervention (Project Respect) to prevent dating and relationship violence.	Not reported.	School staff (*n* = 17), including headteachers, deputy headteachers, and coordinators, as well as a trainer from the National Society for the Prevention of Cruelty to Children.	Intervention fidelity was variable, as some components were not fully implemented. Acceptability was not high, being favored by fewer than two-thirds of staff. Engagement with the material varied among staff, and there was low adherence to policy review and changes in school patrols. Insufficient time was allocated for collective preparation of the intervention, indicating that a trial of the next phase is not immediately warranted.	There was low questionnaire response rates, limiting the analyses, and inconsistent completion of log diaries by staff, hindering the fidelity assessment. The focus on a single health issue may have contributed to the implementation challenges within the school. It is suggested that future research adopts a broader approach and provides greater support to teachers and management staff.	Some concerns (RoB 2)
Millanzi et al. (2022) Tanzania	Quantitative, randomized controlled trial (RCT) intervention design.	To design and test the effect of reproductive health teaching materials within a problem-based pedagogy (PBP) to strengthen socio-emotional skills and promote safe sexual behaviors among adolescents in Tanzania.	Sexual Risk Behavior Beliefs and Self-Esteem Scale (SRBBSES), adapted from Tight et al. (2009) and Unis et al. (2015).	Adolescents aged 12 to 19 (*n* = 660).	The study found that students exposed to reproductive health classroom materials delivered via PBP demonstrated improved socio-emotional skills for safe sexual behaviors, suggesting the integration of these materials and the approach. Post-test results indicated intentions to not consent to anyone wanting to have relationships or sex and to maintain this decision in their daily lives. Evidence suggests the classroom materials can be used as a formal guide for teachers and/or professionals for learning.	The results cannot be generalized to other educational levels as the study focused on secondary schools. The COVID-19 pandemic caused delays in the follow-up phases due to school closures. The lack of recordings or direct observation of how the CSE classes were conducted made it impossible to ascertain the specific content that was delivered. Additionally, the evaluation was conducted by the same individuals who implemented the study, which may introduce bias into the results.	Some concerns (RoB 2)
Mukanga et al. (2024) Zambia	Evaluative qualitative case study design	To analyze the development process of CSE, its quality, mechanisms of change, and the contextual factors influencing its implementation.	Interviews based on Medical Research Council Guidelines and European Expert Group Guidelines for evaluating holistic CSE interventions, covering aspects such as age-appropriateness; gender sensitivity in CSE; student engagement; training and skills in CSE; and interactive teaching. Focus groups to explore experiences in greater depth, alongside direct classroom observation.	Adolescents aged 16–20 (*n* = 35). Teachers (*n* = 17), policymakers (*n* = 4), parents/caregivers (*n* = 4), and religious leaders (*n* = 4).	A discrepancy was found between the guidelines and the actual implementation of CSE, as it is not prioritized or included. Furthermore, there was no linkage between sexual and reproductive health services and CSE in schools, which is crucial for ensuring student access to these services. Due to cultural factors, gender and sexual diversity were not emphasized during lessons. It was noted that the personal opinions of teaching staff compromised the quality of education, as did the lack of adequate training for them.	It was reported that some students and teachers, due to the sensitive nature of the topic, may have provided biased and inaccurate responses. The study recommends better guidance for school staff regarding the curriculum to be adopted.	Score = 14 (Standards for reporting qualitative)
Nuttall et al. (2022) France	Non-randomized, quantitative intervention study with a quasi-experimental design.	To evaluate the effectiveness of the Service Sanitaire (SeSa) program in increasing knowledge of both health students and the adolescent population in Reproductive and Sexual Health education.	A questionnaire comprising 30 true-or-false questions for health students and adolescents, covering: STIs, French laws on Reproductive and Sexual Health (access to abortion, free contraception, laws against homophobia, and access to pornography), and reproductive biology. Risk perception was assessed through the evaluation of situations related to the perception of pregnancy and the risk of STI transmission during first sexual intercourse. Information on sexual behavior was collected using an adapted version of the Centers for Disease Control and Prevention’sYouth Risk Behavior Survey (Kann et al., 2018).	Adolescents aged 13–15 (*n* = 292) and health students (peer educators) (*n* = 747).	The program was effective in increasing knowledge about sexual and reproductive health among both high school and health students, indicating that peer-led programs facilitate informal exchanges and enable more interactions compared to those with teachers. No evidence of impact on behavior was found, as risk behaviors remained unchanged three months post-intervention, an expected outcome given the short duration of the study.	The absence of data matching, which could have ensured greater representativeness of both groups in the pre- and post-tests. The four-month period was too short to assess behavioral impact in adolescents, suggesting a longer follow-up period for future studies. The authors discuss the potential benefit of combining this type of program with social media support for the youth population.	Moderate risk (ROBINS-I)
Orchowski et al. (2023) USA	Quantitative, randomized controlled trial (RCT) design.	To evaluate the impact of a sexual violence prevention intervention for high school students called Your Voice Your View (YVYV).	Questionnaires based on prior research (Coker et al., 2015) assessing experiences of sexual victimization and perpetration of sexual aggression.	Tenth-grade adolescents (*n* = 2685).	A reduction in rates of unwanted sexual intercourse was observed at the six-month follow-up, suggesting that effects may be delayed, indicating the potential for broader applications aimed at increasing protective factors over time. Booster sessions are recommended, consistent with existing literature. The combination of different approaches, such as changing social norms and bystander intervention, was beneficial and well-accepted in the school environment, engaging various social actors.	The methodology for assessing initial and subsequent responses was limited, preventing individual-level analysis. The small number of participating schools (26) is noted, as is the non-participation of some schools in later phases. Furthermore, having one session exclusively for boys may be perceived as a negative aspect, as it excludes non-binary students. Replication of the program is suggested.	Some concerns (RoB 2)
Ponsford et al. (2024) England	Theory-informed qualitative study design.	To examine how well school leaders report that mandatory Relationships and Sex Education (RSE) has been implemented in English secondary schools.	Not reported.	School staff (*n* = 25) responsible for sexual health education.	Despite being mandatory, implementation varied between schools. Classes were scheduled but often taught by tutors or teachers according to gaps in their timetables, with training for these staff being mostly internal and informal. Schools valued student feedback and were concerned with improving the content delivered to students.	Schools self-selected to participate, which may indicate a greater pre-existing inclination towards these issues. The analysis is focused on staff interviews and does not include student perspectives, suggesting this should be incorporated in future interventions.	Score = 18 (Standards for reporting qualitative)
Rasberry et al. (2022) USA	Quantitative, quasi-experimental evaluation study design.	A school-based CSE intervention aimed at school districts, designed to teach essential knowledge and skills to youth.	Student knowledge was assessed using a 50-item instrument administered via Scantron before and after the intervention. Nine items were targeted at high school level, covering topics such as abstinence, personal health, sexual health, STIs/HIV, and pregnancy prevention. Behavioral assessment was conducted using the Youth Risk Behavior Survey, with measures related to sexual behaviors and experiences; analyses concerning physical and sexual violence were restricted to experiences within relationships.	Adolescents (*n* = 7555) from middle and high schools.	The intervention was associated with increased student knowledge about sexual health, greater adoption of protective sexual behaviors, and reduced risk behaviors and experiences. Focusing on dating violence, students showed higher probabilities of reporting forced sex, which contrasted with national data trends. One hypothesis is that the intervention increased awareness and the ability to recognize and report experiences of sexual violence.	The reliability and validity of the measures are unknown as they were developed for educational purposes. There was inadequate control for factors that could impact learning. Only a subset of students analyzed in 2015 had received the intervention, making group comparisons difficult. Additionally, the sample was not representative of the state as a whole.	Score = 7 Low risk (ROBUST)
Shankar et al. (2020) India	Quantitative, quasi-experimental evaluation/survey study design.	Evaluation of the effectiveness of a school-based health education intervention for adolescent girls called Girls Health Champions.	Questionnaires to assess aspects of nutrition and anemia, mental health/gender-based violence, menstruation, and reproductive health. Attitudes were assessed through six questions focused on measuring agreement with health-positive attitudes, with statements such as: “Victims of physical or sexual violence are never to blame or responsible.”	Adolescent girls aged 12 to 16 (*n* = NI).	The study highlights immediate positive impacts on knowledge levels and attitudes across different health domains, which remained consistent throughout the school year. This effect was observed in both peer educators and non-peer educator participants. Some attitudes appeared more difficult to change, as midway through the year only 34% of girls agreed that “Victims of physical or sexual violence are never to blame or responsible,” compared to 31% at baseline.	The absence of a control group means the study cannot attribute changes solely to intervention, suggesting its use in future studies. Attrition of adolescents who tend to have higher scores at the five-month post-assessment follow-up is noted. It is therefore recommended to use individual identifiers to track the characteristics and performance of students who drop out of the program.	Score = 5 Moderate risk (ROBUST)
Tavrow et al. (2023) USA	Non-experimental, quantitative evaluation/survey study design.	To test the validity and feasibility of a student-centered approach using an online instrument to monitor the implementation of state standards for sexual health education.	A student questionnaire covering aspects of contraception and consent, misconceptions about HIV, gender stereotypes and sexual orientation, sexual health services and rights, and sexual harassment, rape, and trafficking. Teacher interviews were also conducted to assess the feasibility of the instrument.	Adolescents (*n* = 515) and teachers (*n* = 5).	Student feedback collected through an online survey was acceptable due to its low cost and valid results. Recently added topics, such as sexual assault and contraception, were less likely to be taught compared to longer-standing mandatory topics like HIV/AIDS. Self-identified LGBTQIAPN+ students gave lower ratings than heterosexual students, indicating a lack of representation of these issues in the curricula.	The significant number of schools that refused to participate. The sample was restricted to 24 high schools with a Gender and Sexuality Alliance club or counselor, meaning schools with such clubs may have differed from those without. The absence of a trained classroom observer also made it impossible to verify the accuracy of student responses.	Score = 5 Moderate (ROBUST)
Whittington (2020) England	Participatory action research qualitative study.	To analyze the outcomes of a participatory action research project on sexual consent with youth.	Group activities and discussions, such as the creation of a Consent Continuum to understand youth perceptions of sexual consent.	understand youth perceptions of sexual consent.A diverse group of youth aged 13 to 25 (*n* = 103), including high school students from a girls-only institution.	The study found that youths’ understanding of consent was based on simplistic legal discourses which, although understood, created discomfort with the dichotomies presented, leading to greater interest in more complex aspects of sexual negotiation. The project successfully deconstructed some socially established norms, with active contributions from youth and high program acceptance.	No specific limitations were reported. The study highlights the need to expand terminologies related to consent and sexual violence to foster greater inclusivity and engagement on the topic.	Score = 15 (Standards for reporting qualitative)

### Implementation contexts

All studies were conducted in school settings. Among the 15 selected studies, most were carried out in the United States (*n* = 4; 26.6%) (Edwards et al., 2023; Orchowski et al., 2023; Rasberry et al., 2022; Tavrow et al., 2023) and the United Kingdom (*n* = 4; 26.6%) (May et al., 2021; Meiksin et al., 2020; Ponsford et al., 2024; Whittington, 2020), which accounted for the largest number of publications. Notably, no studies conducted in Brazil were identified within the defined eligibility criteria.

### Participants

A predominance of female participants was observed in seven studies (46.7%), including one conducted exclusively in a girls’ school. Only the studies by Berutich et al. (2022) and Mukanga et al. (2024) reported a majority of male participants, while three studies (20%) included transgender or non-binary participants (Orchowski et al., 2023; Tavrow et al., 2023; Whittington, 2020). Participants’ ages ranged from 11 to 18 years, considering that age criteria for upper secondary education vary between countries. However, most publications (*n* = 10; 66.7%) focused on adolescents aged 14 to 17. It is noteworthy that none of the analyzed studies examined the intersectionality of gender and race.

### Perspectives included

The studies encompassed a diversity of perspectives. School staff, such as teachers and/or principals, were included in five articles (33.3%); among these, three studies combined their perspectives with those of adolescents (Agu et al., 2024; Mukanga et al., 2024; Tavrow et al., 2023), while two studies examined only professionals’ perspectives (Meiksin et al., 2020; Ponsford et al., 2024). Parents or caregivers participated in only one study (6.6%; Mukanga et al., 2024). Additionally, two studies (13.3%) incorporated other social actors in partnership with the school, such as community leaders, religious leaders, and policymakers (Agu et al., 2024; Mukanga et al., 2024). However, most publications (*n* = 7; 46.6%) examined only students’ perspectives regarding the interventions (Berutich et al., 2022; Gómez-Lugo et al., 2022; May et al., 2021; Millanzi et al., 2022; Orchowski et al., 2023; Rasberry et al., 2022; Shankar et al., 2020).

### Study designs

The studies employed different methodological designs. Only four (26.6%) used qualitative approaches: May et al. (2021) used a phenomenological perspective; Mukanga et al. (2024) conducted a case study; Ponsford et al. (2024) applied a theory-informed evaluation approach; and Whittington (2020) conducted participatory action research. Among quantitative studies, six (40%) employed descriptive and/or survey designs aimed at measuring intervention outcomes (Agu et al., 2024; Berutich et al., 2022; Edwards et al., 2023; Rasberry et al., 2022; Shankar et al., 2020; Tavrow et al., 2023). Another four articles (26.6%) were randomized controlled trials (Gómez-Lugo et al., 2022; Meiksin et al., 2020; Millanzi et al., 2022; Orchowski et al., 2023), while only one study (6.7%) was a non-randomized clinical trial (Nuttall et al., 2022). Fewer than half of the quantitative interventions (*n* = 6; 40%) included follow-up assessments. Only two studies included long-term follow-up: Rasberry et al. (2022), which assessed outcomes three years post-intervention, and Edwards et al. (2023), which conducted five waves of data collection.

### Instruments used

A variety of instruments were used to assess knowledge related to CSE and sexual violence. Most studies employed questionnaires (*n* = 9; 60%) (Agu et al., 2024; Berutich et al., 2022; Edwards et al., 2023; Gómez-Lugo et al., 2022; Nuttall et al., 2022; Orchowski et al., 2023; Rasberry et al., 2022; Shankar et al., 2020; Tavrow et al., 2023). Three studies (20%) used interviews (May et al., 2021; Mukanga et al., 2024; Tavrow et al., 2023), and two (13.3%) applied standardized scales (Gómez-Lugo et al., 2022; Millanzi et al., 2022). One study (6.7%) conducted group discussions through focus groups (Mukanga et al., 2024), and another (6.7%) proposed group-based activities, such as developing a spectrum of different levels and/or types of consent (Whittington, 2020).

### Theoretical frameworks

The included studies drew on diverse theoretical frameworks. Among international guidelines, the UNESCO International Technical Guidance on Sexuality Education (Mukanga et al., 2024) and the World Health Organization Sexual and Reproductive Health Model (Nuttall et al., 2022) was notably present. Only Social Learning Theory (Bandura) was present in more than one study (*n* = 2; 13.3%) (Gómez-Lugo et al., 2022; Shankar et al., 2020). Other frameworks included the Madrid Consensus Document on Evidence-Based Sex Education (Berutich et al., 2022) and the HealthSmart Curriculum, a commercially available program aligned with U.S. health education standards (Rasberry et al., 2022). Additional theoretical models included the General Theory of Implementation (Ponsford et al., 2024), Diffusion of Innovations Theory (Edwards et al., 2023), the Information-Motivation-Behavioral Skills Model for AIDS preventive behavior (Gómez-Lugo et al., 2022), Theory of Planned Behavior and Social Development Model (Meiksin et al., 2020), Problem-Based Pedagogy (Millanzi et al., 2022), the Situational Model of Bystander Behavior, the Integrated Model of Sexual Assault and Acquaintance Rape, and the Assess, Acknowledge, Act Model (Orchowski et al., 2023).

Interventions grounded in explicit theoretical frameworks, such as the Social Learning Theory and Information-Motivation-Behavioral Skills Model tended to demonstrate more consistent effects on socio-emotional skills including self-efficacy and sexual assertiveness (Gómez-Lugo et al., 2022) as well as on protective behaviors and health-positive attitudes (Shankar et al., 2020), whereas studies lacking explicit theoretical grounding more commonly focused on knowledge gains alone (May et al., 2021; Meiksin et al., 2020).

### Content and format of the interventions

The content of the interventions encompassed multiple dimensions: (a) prevention of STIs/HIV; (b) sexual violence, focusing on consent, rape, harassment, grooming, and abusive relationships; (c) contraceptive methods; (d) gender, gender-based violence, and masculinities; (e) puberty and reproductive health; (f) socioemotional skills such as assertiveness, sexual negotiation, self-efficacy, and reporting abuse; (g) information technology, including sexting, pornography, and online grooming; and (h) abortion, considering local legislation. Some studies addressed sexual violence indirectly, such as by promoting assertiveness in relationships (Gómez-Lugo et al., 2022) or fostering more egalitarian relationships (Berutich et al., 2022). In Rasberry et al. (2022), although relationship violence questions were included in the survey, prevention strategies were not part of the intervention content.

Sexual and gender diversity was not examined in 80% of the studies (*n* = 12), and in some cases, this omission was attributed to religious resistance (Mukanga et al., 2024). Intervention formats varied widely in session duration: six studies (40%) offered sessions lasting 30–60 min; two studies (13.3%) exceeded 60 min; and one intervention lasted up to 5 min, integrated into regular classes (Mukanga et al., 2024). Notably, 40% (*n* = 6) of studies did not report session duration or dosage.

### Main outcomes

The reported outcomes were predominantly positive. As several interventions assessed multiple domains (knowledge, attitudes, socio-emotional skills, behaviors), individual studies may appear in more than one outcome category. Whenever explicitly reported, the original distinction between primary and secondary outcomes was preserved. However, because this hierarchy was inconsistently described across studies, outcome classification reflects the domains reported in each study rather than mutually exclusive groupings. In 80% of the studies (*n* = 12), an increase in participants’ knowledge was observed, including greater understanding of STIs/HIV, pregnancy, myths about sexuality, sexual and reproductive health, consent, and recognition of grooming situations. These effects were found in both quantitative studies (questionnaires) and qualitative approaches (interviews and focus groups). Edwards et al. (2023) reported an increase in discussions about sexual violence prevention, although they did not measure STI or sexual violence knowledge directly, similar to Mukanga et al. (2024) and Whittington (2020).

Only 20% of publications (*n* = 3) documented statistically significant reductions in sexual violence indicators, operationalized through distinct outcomes: increased formal reporting and help-seeking following exposure to risk situations (Millanzi et al., 2022); fewer non-consensual sexual encounters (perpetration/victimization) (Orchowski et al., 2023); and reduced relationship violence (victimization) (Rasberry et al., 2022). The remaining studies either did not assess this outcome or found no statistically significant effects (Edwards et al., 2023; Nuttall et al., 2022), as they primarily focused on implementation processes and/or feasibility rather than on direct reductions in sexual violence.

Regarding attitudinal change, nine studies (60%) reported improvements such as greater receptivity to condom and contraceptive use, increased perception of sexual health risks, stronger intentions to refuse forced sex, reduced gender-related stigma, improved recognition of sexual consent, and decreased victim-blaming. In quantitative designs, these improvements corresponded to statistically significant effects, whereas qualitative studies described perceived shifts in attitudes and perceptions. However, two studies (13.3%) identified negative effects: persistence of victim-blaming among some participants (May et al., 2021) and feelings of exclusion reported by LGBTQIAPN+ students (Tavrow et al., 2023).

Consistent with this finding, most interventions did not include content explicitly addressing sexual and gender diversity. Specifically, 80% of the studies (*n* = 12) made no reference to LGBTQIAPN+ identities or experiences. The absence of content addressing sexual and gender diversity was mirrored by the lack of outcome measures specific to LGBTQIAPN+ adolescent). No study examined differential impacts for sexual or gender minority youth.

In terms of socioemotional skill development, 46.6% of publications (*n* = 7) described improvements such as enhanced ability to recognize risk situations and signs of violence in qualitative reports (May et al., 2021), improved communication and negotiation skills (Rasberry et al., 2022; Whittington, 2020). Quantitative studies reported a statistically significant increase in assertiveness (Gómez-Lugo et al., 2022; Millanzi et al., 2022) and in self-efficacy (Millanzi et al., 2022; Shankar et al., 2020) regarding sexual health and sexual violence prevention.

Protective behaviors were also observed in 40% of the studies (*n* = 6). In quantitative evaluations, these outcomes corresponded to statistically significant increases in condom use (Gómez-Lugo et al., 2022; Rasberry et al., 2022; Shankar et al., 2020) and greater health service-seeking behavior (Shankar et al., 2020). However, in some cases, such as Edwards et al. (2023) and Orchowski et al. (2023), increased discussion of these topics did not translate into concrete behavioral change. Finally, 86.6% of the studies (*n* = 13) acknowledged methodological limitations, including lack of follow-up, small sample sizes, insufficient session time, lack of teacher training, focus exclusively on heterosexual relationships, and exclusion of sensitive topics due to cultural barriers.

### Risk of bias assessment

Overall, risk-of-bias assessments indicated considerable variability across studies: randomized controlled trials generally presented “some concerns” or “high risk,” non-randomized trials frequently showed “moderate” or “serious” risks, and survey designs tended to fall within the moderate-risk range according to ROBUST. Qualitative studies received higher SRQR scores, suggesting lower methodological bias.

## Discussion

The scientific production on CSE articulated with the prevention of sexual violence against adolescents in school settings has increased internationally over the years. Although the number of eligible studies in this review was relatively small (*N* = 15), this finding reflects the limited availability of interventions that explicitly integrate CSE and sexual violence prevention for upper secondary students. In addition, this finding aligns with those of previous systematic reviews (Niland et al., 2024) and the prevention of sexual violence among adolescents (Che Yusof et al., 2022). Despite the growth in publications, a significant gap remains: no studies were found in Latin America, and consequently none in Brazil. This suggests the absence of systematic investigations that integrate CSE and sexual violence prevention in these contexts. However, this apparent absence should be interpreted cautiously. It may reflect a true gap in program development or evaluation in the region, but may also stem from limitations in database indexing or publication bias affecting non-English language research. These possibilities underscore the need for more regionally grounded investigations and improved visibility of Latin American scholarship in international databases.

Although the volume of publications has increased, the articulation between CSE and sexual violence remains underexplored. Only 6 of the 15 studies (40%) addressed sexual violence explicitly framing it as a central construct of the intervention. Most studies (*n* = 9; 60%) did not examine sexual violence directly, approaching the topic indirectly using alternative labels such as sexual assertiveness (Gómez-Lugo et al., 2022) or limiting their focus to relationship violence (Rasberry et al., 2022). This pattern contrasts with findings from the epidemiological literature, which indicates that most sexual assaults occur in the domestic sphere, with parents as the main perpetrators (Malta et al., 2025; Mathews et al., 2024). This finding may reflect persistent taboos surrounding sexuality and the cultural and political barriers that hinder its discussion in schools, particularly in the face of advancing conservative agendas (Santos et al., 2021).

Epidemiological data highlight the predominance of intrafamilial sexual violence. However, school-based interventions are primarily directed toward primary prevention, aiming to modify social norms, raise awareness, and strengthen adolescents’ socioemotional competencies before violence occurs (WHO, 2010). Accordingly, sexuality education programs can promote recognition and help-seeking, but they do not replace the protective systems needed to interrupt abuse occurring within households (WHO, 2010).

Gender should be understood not merely as a demographic characteristic but as a structural, macrosocial determinant that shapes both vulnerability to sexual violence and the scope and effectiveness of preventive interventions (WHO, 2010). The predominance of female participants in the analyzed studies aligns with data from UNICEF and the Brazilian Public Security Forum (2021), which indicate that nearly 80% of sexual violence victims are girls, a proportion that surpasses 90% among adolescents aged 15 and older. It should be noted that these figures reflect reported or notified cases, which may underestimate victimization among boys due to cultural norms that influence their self-perception, resulting in difficulties in recognizing men as victims and in expressing vulnerability. Therefore, gender disparities must be interpreted with caution (Ferreira et al., 2023).

Although most included interventions were framed as universal programs, several of them focused disproportionately on girls or implicitly conveyed the assumption that potential victims bear responsibility for preventing violence. As argued by Beres (2014), the understanding of consent varies considerably, and its use in sexual violence prevention is limited. Such approaches may reinforce gendered expectations and perpetuate forms of victim responsibilization, particularly when structural determinants, such as patriarchal norms, unequal power relations, and socialized male entitlement, remain unaddressed. Therefore, interventions designed for girls only or previously victimized adolescents should be understood within a selective or indicated framework, whereas universal programs should explicitly address the social conditions that enable perpetration (Beres, 2014; Michau et al., 2015). A prevention logic grounded in gender equity requires shifting the focus from potential victims to the broader sociocultural norms that shape consent, aggression, and relational dynamics among adolescents. As demonstrated by critical scholarship on consent, presuming that adolescents are universally ignorant about sexual communication, and that the solution lies solely in teaching explicit verbal scripts, can inadvertently perpetuate an “epistemology of ignorance” (Beres, 2021). This is particularly problematic for LGBTQIAPN+ youth, whose sexual negotiations often occur outside dominant heteronormative scripts.

Importantly, while several interventions included male participants, very few studies assessed outcomes related to the perpetration of sexual violence. Only Orchowski et al. (2023) explicitly evaluated both victimization and perpetration, whereas most studies involving boys focused on knowledge acquisition, attitudinal change, socio-emotional skills, or bystander behaviors. Nevertheless, the literature also underscores the importance of investigating boys, given that men who experienced sexual abuse in childhood or adolescence are up to 14 times more likely to perpetrate violence against intimate partners (UNICEF & Brazilian Public Security Forum, 2021). Targeting preventive initiatives toward adolescent boys is also crucial to reduce the perpetration of sexual violence against girls or boys in adolescence and against partners in adulthood (Jewkes et al., 2015). Broadening the focus to different groups of adolescents may contribute not only to protecting girls but also to preventing the perpetuation of violence among boys.

Although prior victimization among boys is associated with a higher risk of later perpetration, this relationship is not deterministic. Rather, it reflects cumulative exposure to harmful gender norms and coercive relational models shaped by broader social contexts, underscoring the need for nuanced interpretations that avoid stigmatizing boys who have experienced violence (Michau et al., 2015; WHO, 2010). It is therefore essential to include adolescent boys in prevention strategies aimed at reducing both immediate and long-term patterns of sexual and relational violence.

A recurring limitation was the absence of intersectional analyses of gender, race, and sexuality. Studies show that non-Hispanic Black adolescents and youth present higher incidence of STIs/HIV and greater vulnerability to intimate partner violence compared with other racial groups (Min et al., 2021). This evidence reinforces that racial inequalities critically shape experiences of sexual health and violence, yet these dimensions remain underexplored in the included studies. Some studies in this review collected race or ethnicity data, but these variables were generally reported only descriptively and were not incorporated into analytical models or stratified outcome analyses. In other cases, such data were entirely absent. Consequently, the intersectional effects of race, gender, and sexuality on vulnerability to sexual violence and intervention outcomes remain largely unexplored. In addition, the exclusion of LGBTQIAPN+ populations was highlighted by Gómez-Lugo et al. (2022), Tavrow et al. (2023), and Mukanga et al. (2024), who noted a lack of curricular representation. Considering that gender, race, and sexuality operate in an intersectional and inseparable manner (Veiga, 2018), this omission undermines the breadth and social relevance of the interventions.

Another relevant aspect concerns community engagement as a facilitator of the success of CSE, albeit a challenging one. UNESCO (2019) indicates that the participation of social actors can strengthen the legitimacy and acceptance of CSE. This dimension was positive in Agu et al. (2024), in which the involvement of community leaders fostered social acceptance of intervention. This finding aligns with previous reviews (Michau et al., 2015) that emphasize that effective violence prevention requires coordinated actions across individual, relational, community, and societal levels, rather than focusing solely on individual behavior change.

Conversely, Mukanga et al. (2024) showed that the influence of religious leaders in conservative contexts resulted in the censorship of content, undermining the effectiveness of the proposal. We hypothesize that changes in students’ attitudes and practices are linked to broader shifts in the school context, hence the value of partnerships with families and communities. These findings suggest that future research must consider strategies to overcome cultural and religious barriers that limit the implementation of CSE.

Methodologically, the included studies used diverse designs, which makes direct comparisons challenging, but provides relevant contributions. In quantitative designs, increases were observed in knowledge about sexual and reproductive health and in the adoption of protective behaviors (Agu et al., 2024; Rasberry et al., 2022), confirming expectations from the international literature (UNESCO, 2020). In some cases, these impacts were maintained over time, as evidenced by Shankar et al. (2020), underscoring the importance of continued interventions. Randomized controlled trials showed efficacy in reducing sexual risk behaviors and strengthening self-efficacy and assertiveness (Gómez-Lugo et al., 2022; Orchowski et al., 2023), whereas the non-randomized trial by Nuttall et al. (2022) showed benefits for students and peer educators. Qualitative studies, such as May et al. (2021), revealed that participatory methodologies, including the use of theater, were effective in raising awareness about sexual abuse, perpetrators, and abusive relationships.

Despite these promising results, the challenge of translating knowledge into behavioral change persists. Studies such as Edwards et al. (2023) and Orchowski et al. (2023) showed an increase in peer discussions but did not find significant reductions in sexual violence behaviors. Prior reviews (Carmo et al., 2025; Rivera et al., 2021) had already highlighted this gap, reinforcing the need to investigate mechanisms that facilitate the transformation of attitudes into concrete practices. One possible explanation is that this transition depends on the strengthening of socioemotional competencies. UNESCO (2019) emphasizes that CSE should include life skills such as communication, negotiation, and assertiveness, which are essential for building healthy relationships. Evidence corroborates this role: Widman et al. (2018) found significant improvements in sexual communication and condom-use self-efficacy, while Gómez-Lugo et al. (2022) identified increased self-efficacy and reduced sexual risk behaviors, with effects maintained up to six months post-intervention.

The predominance of self-report instruments, such as questionnaires, allowed the measurement of acquired knowledge (Gómez-Lugo et al., 2022; Millanzi et al., 2022), but constrained the understanding of more complex processes. Qualitative methods, such as interviews and focus groups (Whittington, 2020), and participatory approaches, such as arts-based methods (Leavy, 2020), can supplement findings, foster greater participant engagement, and enhance the validity of interventions.

Another critical point concerns the theoretical underpinnings and implementation of interventions. Although national and international guidelines mandate CSE, its inclusion in school curricula remains fragmented, lacking standardization and sufficient teacher training (Ponsford et al., 2024). In some cases, personal and cultural factors among school personnel compromised intervention quality, resulting in the suppression of sensitive content (Tavrow et al., 2023) or low institutional adherence (Meiksin et al., 2020; Mukanga et al., 2024). These barriers underscore that social resistance to openly discussing sexuality and sexual violence remains a significant obstacle to implementing CSE (Hailes et al., 2019; Santos et al., 2021).

These limitations also reflect gaps in teacher training. Higher education rarely includes debates on sexuality, which reinforces restrictive medical or religious perspectives (Rodriguês et al., 2021). Successful interventions, such as the development of formal guides (Millanzi et al., 2022) and the training of school professionals (Agu et al., 2024), demonstrate pathways to strengthen teacher preparation and increase school adherence.

Sampling problems were also recurrent. In qualitative studies, there was a lack of systematic selection strategies and recruitment difficulties. In randomized controlled trials and in the non-randomized study (Nuttall et al., 2022), questions arose regarding randomization quality and participant adherence (Meiksin et al., 2020). As Cozby (2003) notes, adequate randomization is important to ensure group comparability and reduce bias. In descriptive quantitative studies, convenience samples predominated, limiting the generalizability of findings (Hultsch et al., 2002; Scholtz, 2021). Moreover, the reliability of the measures used was rarely reported, an exception being Rasberry et al. (2022), which compromises methodological robustness (Gremigni, 2020).

Finally, the absence of limitation statements in some articles (Berutich et al., 2022; Whittington, 2020) undermines scientific transparency. As argued by Clarke et al. (2023), explicitly reporting methodological weaknesses is essential to advance the field and guide future research. Likewise, the absence of conflict-of-interest declarations in four studies (Edwards et al., 2023; May et al., 2021; Orchowski et al., 2023; Rasberry et al., 2022) poses ethical concerns, since transparency is essential for academic credibility (Ventura & Oliveira, 2022).

One limitation of this review concerns access to full-text articles. Although searches were conducted across multiple databases, retrieval of full texts depended on institutional access through the researchers’ affiliated universities. Importantly, no potentially eligible studies were excluded solely due to lack of access to full texts. Nevertheless, it is possible that some relevant studies beyond these access networks were not retrieved, which could have influenced the comprehensiveness of the evidence base. Nonetheless, the transparent search process and adherence to PRISMA guidelines (Page et al., 2021), enhance the methodological rigor of the review. Although a broad and conceptually driven search strategy was used, the reliance on a limited set of English descriptors may have restricted the retrieval of studies employing alternative terminology, potentially leading to the omission of some relevant interventions. Likewise, Portuguese-language searches were restricted to SciELO and BVS, which may have resulted in the underrepresentation of relevant Lusophone studies indexed in other regional or international databases, potentially introducing language or regional publication bias.

Additionally, another limitation of this review concerns the temporal cutoff established from 2019 onwards. Although this decision was grounded in major international policy milestones, it potentially resulted in the exclusion of earlier foundational interventions, thereby constraining historical comparability with previous studies. Consequently, important developments in CSE and sexual violence prevention prior to this period might not have been fully captured.

Overall, the findings of this review are consistent with prior evidence that interventions in CSE and sexual violence prevention support healthy sexual development in adolescence by enhancing self-knowledge, preventing STIs/AIDS, and strengthening socioemotional skills (Bragg et al., 2021; Che Yusof et al., 2022; Niland et al., 2024). By expanding self-perception and the capacity to recognize and confront abusive situations (May et al., 2021), these initiatives help break cycles of violence. However, only 20% of the studies provided evidence of changes in sexual-violence indicators. Our findings do not clarify the extent to which these results derive from shortcomings in the design, implementation, or evaluation of interventions. Nevertheless, the lack of follow-up identified in many studies may impede the detection of complex behavioral changes.

Therefore, the core contribution of this work lies in delineating the structural limitations and operational disconnects inherent in existing programs. Through its analytical mapping, this review establishes an evidence-based framework to guide the development of more coherent and effective interventions, thereby moving the field beyond fragmented or purely biological paradigms. These insights reinforce the need for public policies and school practices to incorporate evidence-based interventions that integrate CSE and sexual violence prevention. To increase effectiveness, programs must be culturally and socially adapted to adolescents’ realities, address gender, racial, and sexual diversity, and involve not only students but also educators, families, and community actors. By adopting this integrated and context-sensitive approach, it will be possible to advance toward more inclusive, legitimate, and sustainable educational practices, ultimately contributing to the promotion of sexual health and the guarantee of adolescents’ human rights in school contexts.

## Conclusion

In response to the guiding question: “How have school-based interventions with upper secondary students been implemented, articulating CSE and the universal prevention of sexual violence?”, this systematic review revealed that such interventions have been implemented predominantly through short-term, curriculum-based programs focusing on students’ attitudes and knowledge. Across studies, consistent and robust effects were observed for knowledge acquisition and attitudinal change. In contrast, evidence for behavioral outcomes and for direct reductions in sexual violence or related victimization was more limited and heterogeneous, reflecting both the short duration of interventions and methodological constraints. These initiatives often lack the engagement of broader school actors, such as teachers and administrators, and show methodological variability, with a predominance of quantitative designs and limited theoretical consistency. Nonetheless, this review highlights that mixed-methods approaches integrating quantitative and qualitative data can provide a more comprehensive understanding of the phenomenon.

The inclusion and characterization of intervention, survey-based, and clinical trial studies expand international knowledge on the state of the art in this field, which has intensified in recent years. However, this expansion has not been mirrored in Brazilian literature, highlighting the need for more research. This apparent absence of Brazilian studies may also reflect limitations related to database coverage, indexing practices, and language restrictions, rather than exclusively indicating a true research gap in the national context. Given this scenario, the findings offer consistent answers to the proposed research question and address a gap identified in prior reviews, by systematically integrating CSE and sexual violence prevention, which have often been examined as separate domains.

The limitations of this review stem partly from publication bias among the identified articles, due to the inclusion criteria adopted. Although direct contact with authors is recommended when full texts are inaccessible, this strategy was not pursued. This decision was based on the small number of unavailable records and the assessment, through available metadata (titles and abstracts), that their inclusion would be unlikely to substantially modify the overall findings.

Despite the generally satisfactory quality of the analyzed publications, relevant methodological limitations were identified, such as the heterogeneity of study designs, which preclude broad generalizations. Future research should pursue greater methodological standardization, particularly in the reporting of intervention dosage, the specification of core outcome domains, the establishment of minimum follow-up periods, and the systematic assessment of implementation fidelity, as well as adopt longitudinal approaches that enable the exploration of causal relationships that remain underexamined.

Notwithstanding the limitations identified, the systematization of evidence proved productive and revealed key directions for research and practice. Based on the findings, we recommend that future investigations invest in probability samples, employ validated instruments, and devote greater attention to the sociocultural contexts of the research, while considering researchers’ reflexivity regarding their own positions and assumptions. Efforts toward the design, implementation, and evaluation of new interventions should consider the potential to produce and detect changes in distal indicators of sexual violence, in addition to proximal attitudinal and behavioral changes. It is further imperative to address dimensions that are often rendered invisible, such as race, social inequalities, and dissident sexualities.

From a public health and educational perspective, these findings suggest that upper secondary students represent a key target group for the universal prevention of sexual violence, given their developmental stage and potential for critical engagement. Schools emerge as privileged settings for implementing evidence-informed interventions capable of promoting sustainable changes in knowledge, attitudes, and potentially behaviors.

Therefore, future efforts should prioritize the development of culturally grounded, longitudinal studies that incorporate multiple stakeholders and address often-overlooked dimensions. By systematically integrating sexuality education and sexual violence prevention, this review advances current knowledge on school-based interventions for adolescents and identifies key empirical, methodological, and conceptual gaps to guide future research and practice. Advancing these areas is essential to strengthen both the scientific evidence base and the effectiveness of CSE and sexual violence prevention strategies during adolescence.

## Supplementary Information

Supplementary Material 1.

## Data Availability

No datasets were generated or analysed during the current study.

## Abbreviations

AIDS: Acquired Immunodeficiency Syndrome
BVS: Virtual Health Library
CSE: Comprehensive Sexuality Education
ECA: Statute of the Child and Adolescent (Brazil)
LGBTQIAPN+: Lesbian, Gay, Bisexual, Transgender, Queer, Intersex, Asexual, Pansexual, Non-binary, and other identities
MEC: Ministry of Education (Brazil)
PCN: National Curriculum Parameters (Brazil)
PICOS: Participants, Intervention, Control, Outcomes and Study designs
PRISMA: Preferred Reporting Items for Systematic Reviews and Meta-Analyses
PROSPERO: International prospective register of systematic reviews
RoB 2: Revised tool to assess risk of bias in randomized trials
ROBINS: I-Risk Of Bias In Non-randomized Studies of Interventions
ROBUST: Risk of Bias Utilized for Surveys Tool
SINAN: Information System for Notifiable Diseases (Sistema de Informação de Agravos de Notificação)
STI: Sexually Transmitted Infection
UN: United Nations
UNESCO: United Nations Educational, Scientific and Cultural Organization
UNICEF: United Nations Children’s Fund
WHO: World Health Organization
