# Supplementary material for: School-based interventions for sexual education and sexual violence prevention in adolescence: systematic review
Source: Psicol Reflex Crit. 2026 Apr 18;39:19. doi: 10.1186/s41155-026-00390-5 (PMC13191010; doi:10.1186/s41155-026-00390-5)
Supplement: Supplementary file 1 — Supplementary Material 1. [file 41155_2026_390_MOESM1_ESM.pdf]

**Appendix A**

Preliminary combinations of keywords and descriptors were pilot-tested across the selected databases. Based on the relevance, sensitivity, and volume of retrieved records, the final search strategy was defined as the combination that yielded the highest number of studies aligned with the objectives and scope of this systematic review.
Initially, the following combinations were tested:

("sex education" OR "sexual health education" OR "sexual prevention" OR "sexual violence prevention" OR "sexual abuse prevention" OR "sexual assault prevention") AND (promotion OR intervention) AND (school)

("sex education" OR "sexual prevention") AND (promotion OR intervention) AND (school)

("sex education" OR "sexual violence prevention") AND (promotion OR intervention) AND (school)

Based on the results of these exploratory searches, the following database-specific strategies were adopted:

Web of Science
("sex education") OR ("sexual violence prevention") OR ("sexual abuse prevention") AND (promotion OR intervention) AND (school)
PsycINFO
("sex education") OR ("sexual violence prevention") OR ("sexual abuse prevention") AND (promotion OR intervention) AND (school)
PubMed
("sex education") OR ("sexual violence prevention") OR ("sexual abuse prevention") AND (promotion OR intervention) AND (school)
DOAJ
("sex education") OR ("sexual violence prevention") OR ("sexual abuse prevention") AND (promotion OR intervention) AND (school)
Scielo
(((*"educação sexual") OR ("prevenção violência sexual")) AND ((promoção OR intervenção) )) AND (escola)
BVS
("sex education") OR ("sexual violence prevention") OR ("sexual abuse prevention") AND (promotion OR intervention) AND (school)
("educação sexual") OR ("prevenção sexual") AND (promoção OR intervenção) AND escola
